# Mapping Above- and Below-Ground Carbon Pools in Boreal Forests: The Case for Airborne Lidar

**DOI:** 10.1371/journal.pone.0138450

**Published:** 2015-10-01

**Authors:** Terje Kristensen, Erik Næsset, Mikael Ohlson, Paul V. Bolstad, Randall Kolka

**Affiliations:** 1 Department of Ecology and Natural Resource Management, Norwegian University of Life Sciences, Ås, Norway; 2 Department of Forest Resources, University of Minnesota, Saint Paul, Minnesota, United States of America; 3 United States Forest Service, United States Department of Agriculture, Grand Rapids, Minnesota, United States of America; University of Maryland at College Park, UNITED STATES

## Abstract

A large and growing body of evidence has demonstrated that airborne scanning light detection and ranging (lidar) systems can be an effective tool in measuring and monitoring above-ground forest tree biomass. However, the potential of lidar as an all-round tool for assisting in assessment of carbon (C) stocks in soil and non-tree vegetation components of the forest ecosystem has been given much less attention. Here we combine the use airborne small footprint scanning lidar with fine-scale spatial C data relating to vegetation and the soil surface to describe and contrast the size and spatial distribution of C pools within and among multilayered Norway spruce (*Picea abies*) stands. Predictor variables from lidar derived metrics delivered precise models of above- and below-ground tree C, which comprised the largest C pool in our study stands. We also found evidence that lidar canopy data correlated well with the variation in field layer C stock, consisting mainly of ericaceous dwarf shrubs and herbaceous plants. However, lidar metrics derived directly from understory echoes did not yield significant models. Furthermore, our results indicate that the variation in both the mosses and soil organic layer C stock plots appears less influenced by differences in stand structure properties than topographical gradients. By using topographical models from lidar ground returns we were able to establish a strong correlation between lidar data and the organic layer C stock at a stand level. Increasing the topographical resolution from plot averages (~2000 m^2^) towards individual grid cells (1 m^2^) did not yield consistent models. Our study demonstrates a connection between the size and distribution of different forest C pools and models derived from airborne lidar data, providing a foundation for future research concerning the use of lidar for assessing and monitoring boreal forest C.

## Introduction

The boreal forest is a vital net sink in the global carbon (C) cycle, being responsible for ~22% of the global residual terrestrial CO_2_ uptake between 1900 and 2007 [1]. The boreal forest zone is of particular interest because it is situated at latitudes undergoing great climatic stress, possibly altering its current role as a C sink [2]. Most studies exploring the role of boreal forests rely on simulations [3, 4], but limited empirical data even in regions subject to intense investigation, result in models with coarse resolution and a high degree of uncertainty. To improve estimates and model accuracies of C stocks and fluxes, more direct observations from local stands are needed. Accurate reporting of the C stocks in forested ecosystems is also a requirement for countries ratifying the Kyoto protocol to the United Nations Framework Convention on Climate Change (UNFCCC). To monitor, report, and verify C stocks, there is a need for repeatable, cost-effective methods to estimate above- and below-ground C components over large areas. Airborne scanning light detection and ranging (lidar) systems can provide quantitative spatial information on forest structures, and have therefore been acknowledged to have a strong potential for monitoring C pools [5]. However, no studies have yet attempted to employ secondary information from lidar, such as canopy gap structure, non-canopy echo densities, and ground echoes (topographical features) to model the C stock in multiple forest compartments.

Airborne lidar has been widely used for forestry purposes, generating data on stem volume [6, 7], canopy height [8–10], and basal area [6, 11]. Because these biophysical properties are closely associated to tree biomass, which can be computed by allometric equations, studies have successfully demonstrated the use of lidar for both above- and below-ground biomass estimates [12]. Thus, tree biomass data derived from airborne lidar can be used to comply with various international conventions requiring reports on C storage in trees. For example, New Zealand uses lidar in an operational forest C inventory system as part of their commitment to the Kyoto Protocol [13, 14].

Boreal forest soils represent a large C pool characterized by high spatial and temporal variations [15, 16], making accurate assessments of soil C expensive and labor intensive. For purposes such as investment planning in forest C offset projects, the variability and rate of soil C storage and accumulation is a major challenge. Because the loss of value is often smaller than the cost of accurate measurements, offset projects regularly ignore potential C credits from this compartment [17]. Of particular interest for C mapping is the organic layer of boreal forest soils, as it is believed that the most rapid and profound changes from a changing climate will occur here, due to the tight coupling between soil processes and properties of vegetation structure [18, 19]. Although lidar cannot provide direct measurements of soil C, it delivers accurate measurements of stand structure properties such as volumetric forest properties [6, 20] and species composition [21, 22], which all have been linked to the organic layer C stocks and fluxes in earlier ecological studies [23–25]. A particularly promising aspect of small footprint laser data, compared to other remote sensing imagery such as optical sensors [26], is the ability to map topographical features with high detail [27]. Local topography have been acknowledged to be a strong predictor for accumulation of C in top soil horizons [28, 29], and surveyors have utilized digital elevation maps (DEM) from remote sensing as auxiliary information in mapping organic layer C [28, 30].

Trees and forest soils have received widespread attention for their role and importance in C cycling, but less is known about the role of the forest understory vegetation. Although usually containing a relatively small portion of the forest C stock at a given time, understory vegetation can produce as much as 10 to 35% of the total annual input of organic litter [31, 32]. Despite being a key component in the forest C cycle [33], it is often left out of forest C models due to the lack of empirical evidence [34]. The productivity of the understory vegetation is strongly associated with light availability, which in turn is a result of the above-ground dynamics such as species composition [35], development phase, and time since last disturbance [36]. Compared to conventional optical remote sensing such as Landsat, returns from lidar have the advantage of mapping three-dimensional surface structures [37], which includes information on surfaces below canopies, such as the understory vegetation. Thus, recent studies have successfully used structural parameters from lidar as proxies for understory light availability [38] and to map understory species abundance and distribution [39–42].

The main objective of the present study was to explore the capability of discrete return lidar to estimate C stocks in living forest compartments and the soil organic layer. In addition, we investigated the scale effects of the relationships between lidar variables and field measurements. To enable a quality assessment of how lidar copes with the natural heterogeneity, all stands used in this study are mature, multilayered boreal spruce forests, which have not been impacted by any form of forest practices in the last century [43].

## Methods

### Ethics statement

The boreal forest plots used in our study was privately owned and was not protected in any way. Permission to conduct the described field studies, involving the collection of field and soil samples was granted by the land owner, Fritzøe Skoger. The sampling did not involve endangered or protected plant or animal species in the study area.

### Study area

**Fig 1:**
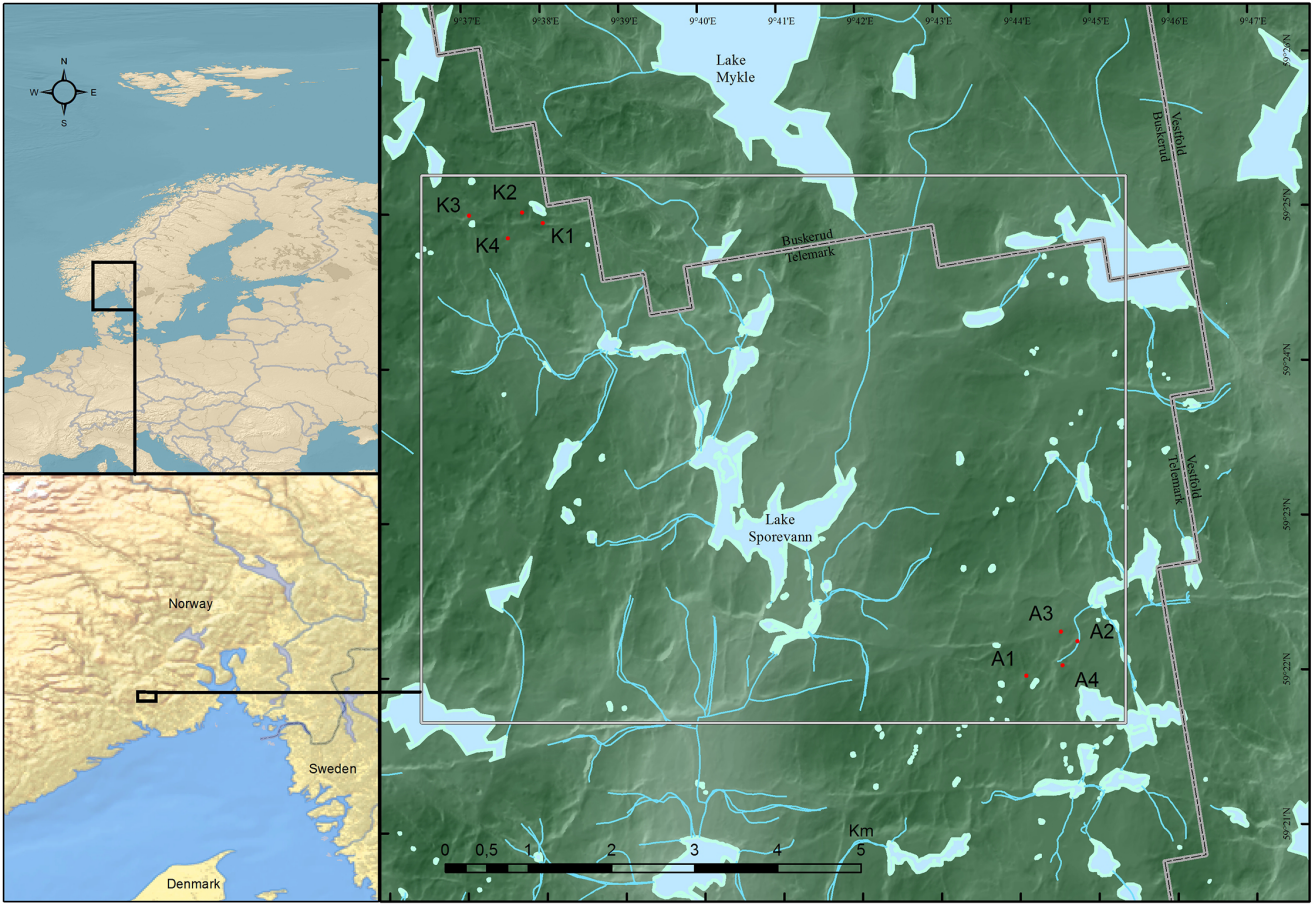
Map of study area. Location of the study plots in a boreal forest landscape in SE Norway. Plots labeled with K are located close to the small lake Kapteinstjern, and plots labeled with A are located SW of the lake Årumsvannet. Reprinted from Kartverket under a CC BY license, with permission from Kartverket, original copyright Kartverket 2013.

Eight circular study plots with a 50 m diameter (~2000 m^2^) were positioned in mature and multilayered spruce forests in the Årum–Kapteinstjern area, located approximately 35 km north of Skien in SE Norway (Fig 1). Six of the eight plots were selected randomly, i.e. four were positioned by random in the forest landscape SW of the lake Årumvannet, and two were randomly positioned adjacent to the forest landscape near Lake Kapteinstjern (Table 1). Here, were also two plots located subjectively to cover the occurrence of the red-listed lichen *Usnea longissima* [44]. The area is considered situated in the border of the south–middle boreal vegetation zone [45]. Climate is oceanic [45], with an annual mean temperature approx. 3.3°C, and average extreme temperatures 14.5°C in July and -7°C in January. Annual precipitation averages 1120 mm, with a high of 115 mm in July, and a low of 60 mm in February. The forests belong to the *Picea*—*Vaccinum myrtillus* type [46, 47], which are the most abundant forest type in NW Europe. The forests are dominated by Norway spruce (*Picea abies* (L.) Karsten), but scattered occurrences of Scots pine (*Pinus sylvestris* L.) and birch (*Betula pendula* Roth and *B*.*pubescens* Ehrh.) are common.

**Table 1:** Background information of the study plots.

	Plot (A = Årum, K = Kapteinstjern)							
	A1	A2	A3	A4	K1	K2	K3	K4
Latitude N	59°36'63'	59°36'99'	59°37'10'	59°36'73'	59°41'55'	59°41'67'	59°41'64'	59°41'39'
Longitude E	9°73'50'	9°74'59'	9°74'24'	9°74'26'	9°63'35'	9°62'92'	9°61'79'	9°62'61'
Altitude (m.a.s.l)	572	516	520	522	643	649	615	641
Aspect	NW	NE	N	N	S	N	S	SE
Stem density (ha^-1^)	351	611	688	652	270	484	479	565
Basal area (m^2^ ha^-1^)	30	26	20	20	24	28	28	17
Stand age (yrs. ±1sd)	115 ±55	121 ±53	146 ±57	143 ±71	190 ±56	183 ±72	155 ±63	188 ±59

The understory vegetation in the area is characterized by European blueberry (*Vaccinium myrtillus*), feather mosses (in particular *Pleurozium schreberi* and *Hylocomium splendens*), green peat moss (*Sphagnum girgensohnii*) and common haircap moss (*Polytrichum commune*). There is usually a distinct height difference between the overstory and understory vegetation in these forests, making these stands suitable for investigating the understory layer separately. The soils in this area are mesic to mesic/moist podzols, nutrient poor with a low pH [48].

### Field data acquisition and preparation

#### Trees

To estimate standing biomass using traditional field methods we divided the trees into two classes, under and above 1.3 m tall. All trees >1.3 m within each plot were measured for diameter at breast height (dbh) using a caliper. Then 20 trees were selected using a relascope, a technique which selects trees with a probability proportional to their basal area [49]. The selected trees were then measured in height using a Vertex hypsometer. Based on the 20 sample trees from each plot, height-diameter regression models were developed for each specific plot and species (Norway spruce, Scots Pine and deciduous spp.):

h=α+dbhβ+ε (1)

where *α* α is the species-specific constant, dbh is the diameter at breast height, and *β* the plot specific regression coefficient, which in these plots ranged from 0.82 to 0.90, *ε* is a normally distributed error term. Basal area was calculated for all trees > 1.3 m.

The most reliable way of determining tree C stocks is through harvest and laboratory analysis, which is a destructive and labor intensive method. Thus, tree biomass and C stocks is commonly projected from species-specific allometric equations [50]. In this study, above and belowground tree biomass were estimated using species-specific allometric models developed from data across Sweden (S1 Table) covering a variety of stand properties like stand age, site index and basal area [51, 52]. To estimate C storage in tree compartments we followed the established practice of assuming 50% C content in tree biomass [53]. Total plot estimates of above- and below-ground tree C stock was computed as the sum of all individual trees within each plot. Tree cores were extracted using an increment borer and individual tree age was later determined in the laboratory by analysing the growth rings.

Young tree saplings < 1.3 m were measured for height, before sixteen randomly chosen saplings on each plot were harvested. After being oven-dried (Thermax Series TS8000) at 65°C to a constant mass, the weights were used to develop a regression model for sapling biomass.

#### Understory vegetation and organic layer

**Fig 2:**
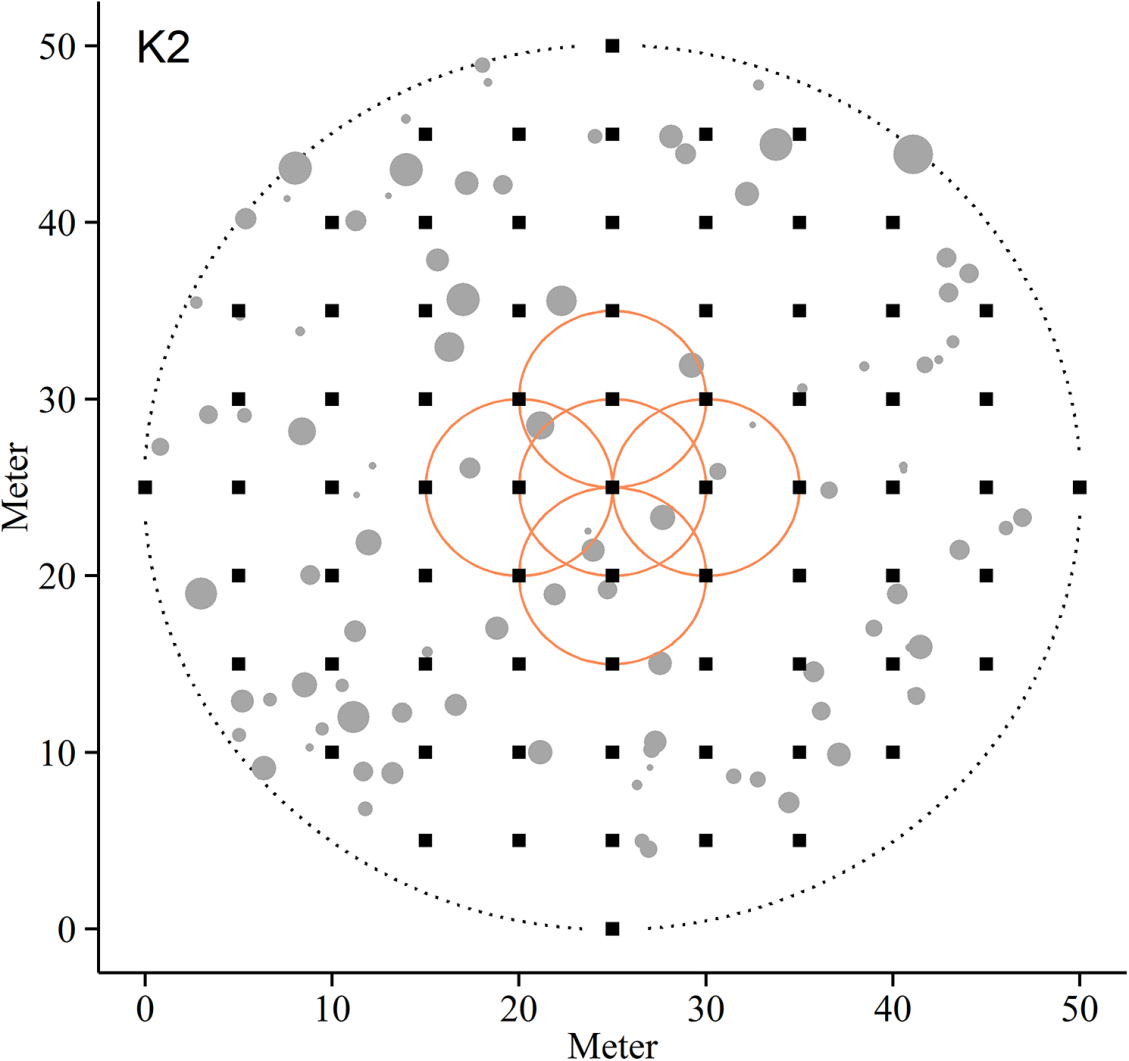
Sampling strategy. Sampling strategy for the eight boreal forest plots used in this study, illustrated by plot K2. Filled squares represent 73 sample points for field layer vegetation, mosses and the organic top soil layer. Filled circles indicate location of standing trees. Stand density measures were calculated at a plot scale (dotted circle) and in subplots with a radius of 5 m around for each of the 73 sampling points (full circles). For illustration purposes only five of the density circles are displayed.

Each plot was divided into a systematic grid containing 73 sampling points, with a distance between each point being 5 m in both north-south and east-west directions (Fig 2). All field layer vegetation (shrubs and herbaceous plants) within a quadrat (625 cm^2^) was clipped at a ground level. The biomass is considered as the field layer compartment from the clip plots. For the purpose of this study, the C stock in saplings is analyzed separately and not as a part of the field layer compartment. We have based the divisions of the different compartments on traditional a priori grouping defined by discrete and measureable biological trait differences [54].

Separate samples of both moss and soil organic layer were collected from the center of the quadrat using a cylindrical steel corer (d = 56 mm). Mosses are considered separately from field layer. However, the term understory encompasses field layer, saplings and mosses. The soil organic layer consists of the F (O_e_) and H (O_a_) horizon, partially decomposed matter and well-decomposed organic matter, down to the mineral soil boundary. The boundary between the organic horizons and mineral soil is sharp and clear visually due to the low faunal mixing of decomposing litter in these forests. Since root biomass is estimated from the above-ground data, living roots (> 2 mm) were excluded from the soil sample to avoid double counting. After collection, all samples were stored in separate paper bags and dried in room temperature (15 to 20°C).

Individual coordinates for each tree and field samples were acquired using two differential global positioning systems (GPS) and global navigation satellite systems (GLONASS) 40-channel dual-frequency survey grade receivers (Topcon Legacy) as field and base units. We established ground truth coordinates for the plot center and each sample collected at the end of centerlines. The distance between the plots and the base station was less than 10 km.

All samples were dried at 65°C in a drying oven until reaching a constant mass. Samples were then weighed again to determine dry content. To determine C concentration (C_c_), samples were grounded to a size of < 100 μm using a ball mill, before the homogenized mixture was analyzed using a VarioMax EL CHN analyzer with a TCD detector (Elementar Analysensyterne GmbH, Hanau, Germany). The analysis was conducted at the Skogforsk (Ås, Norway) commercial laboratory and complied with ISO 9000 certified methods. The stock of C was estimated by multiplying the sample weight of the organic material (per unit area) with the C_c_ derived from the sample material.

### Lidar data acquisition and processing

A Piper PA31-310 aircraft flying approximately 500 m above ground carried the ALTM 3100C (Optech, Canada) scanning lidar system. The pulse frequency was 100 kHz with an average pulse density of 4.5 (± 0.8) m^-2^. Pulses with scan angles exceeding 15° were excluded from the data. After lidar data acquisition, standard filtering procedures were used [55] considering only the first and last echoes. Planimetric coordinates and ellipsoidic heights were computed from processing first and last echoes. Ground echoes were extracted from the last echo data by filtering out local maxima representing echoes from vegetation. From the planimetric coordinates and height values of each ground point retained from the last echo data, a digital elevation model (DEM) was rendered using a thin plate spline interpolation on 1m grid cells. The smoothing parameter (*λ*) for the interpolation was determined by generalized cross validation [56]. To avoid any edge effects in the DEM, the interpolation was conducted on a dataset including ground returns up to 10 m outside the plot window. The expected ellipsoidic height accuracy of the DEM is approximately 25 cm [12].

For estimation of the living C stock (trees and understory vegetation), first and last echoes were spatially registered to the DEM according to their coordinates. The height of each individual echo (point) was then computed by subtracting terrain surface height from the first echo height. Because first echoes from tree canopies have shown to be more stable than last echoes across different lidar configurations and flying heights [55], we only used first echoes in the tree biomass estimates. However, for understory biomass predictions, both first echoes and combined first-last echoes were considered. Non-ground echoes from outside the plot windows were excluded from further analysis and lidar metrics were aggregated in bins representing each plot.

### Statistical analysis of field data

#### Field data characteristics

All statistical analyzes were carried out with the software package R, version 3.0.2. [57]. Standard statistical methods were used describe central trends and spread, without considering the spatial nature of the data. One-way analysis of variance (ANOVA) was used to determine if there were any statistically significant differences between means or distributions. Statistical significance was accepted at *α* = 0.05 level. Relationships between parameters were described using Pearson’s, Spearman rank correlation coefficient or least squares regression.

#### Modeling field data

After determining the spatial properties the stems, each sample point was tested against a number of point measurements relating to the spatial configuration of stems. A nearest neighbor distance analysis was used to determine the distance from each of the sampling points to the surrounding trees. After proximity was determined we computed stem density (n m^-2^), basal area density (m^2^ m^-2^) and above-ground biomass density (kg m^-2^) in a radius of 5 m from each of the sampling points (Fig 2). As sampling points close to the plot boundaries can be affected by trees outside the observation window, the nearest neighbor analysis was also used to identify points positioned closer to the plot window than the given radius. These samples were excluded from further analysis to avoid errors caused by edge effects. Tree density data was evaluated against each C compartment on all eight plots using a linear regression model.

### Lidar metrics

#### Above-ground echoes

A total of 83 vegetation metrics were derived from the lidar echoes and divided into two categories, above and below 1.5 m, representing the overstory and understory vegetation (S2 Table). From the overstory echoes standard metrics of overstory canopy heights (h) were computed. Canopy densities (cd) were computed as the proportion of first echoes for ten intervals representing proportions of echoes from the lower canopy limit (1.5 m) up to the 95^th^ percentile, including the mean, maximum, standard deviation and coefficient of variation (CV) [58]. Since understory conditions, such as light admittance, not only depends on the canopy right above the point of interest, we computed additional canopy densities for each plot containing buffer areas from 2 to 10 m (in 2 m intervals) outside the original plot window. Although data on the canopy density distributions are useful for individual plot measurements, they are not as well suited for direct comparisons between plots. To evaluate differences in canopy characteristics between plots, we therefore computed canopy distribution for five fixed stratum bin heights (S2 Table).

To explore the use of lidar data in mapping and quantifying understory biomass we computed understory metrics using both combined echoes and first echoes only. Two filters were applied; Filter1: including all non-ground echoes up to 1.5 m, and Filter 2: non-ground echoes 0.2 m to 1.5 m. From these four datasets (combined echoes using filter 1, combined echoes using filter 2, first only using filter 1 and first only using filter 2) we first estimated the understory intensity (U_i_) as the proportion of non-overstory echoes above the given threshold. U_i_ is given as a fraction of the effective area covered divided by the total plot area. Second, metrics of understory heights (U_h_) which includes quantiles equivalent to the 0, 10,…90^th^ percentiles, mean, maximum, standard deviation and coefficient of variation were computed. Third, the distribution of understory echoes was classified in three separate height bins (Filter 1: 0 to 0.5 m, 0.5 to 1 m, 1 to 1.5 m, Filter 2: 0.2 to 0.5 m, 0.5 to 1 m and 1 to 1.5 m).

To further inspect the relation between overstory and understory biomass, we delineated the area into canopy or canopy interspace at 1 m^2^ raster resolution, classifying each cell depending on if the cell contained echoes from the overstory or not. Pixel-based class information was then mapped with the field sampling points to determine the sample class (canopy or canopy interspace) of each individual sample. Classification of sampling points was controlled by using a nearest neighbor analysis to determine distance to nearest stem. Points were excluded from the canopy analysis if: (1) classified as canopy interspace according to lidar canopy maps, but located <0.45 m (-2sd) from the nearest stem, or (2) classified as under canopy, but located ≥4.9 m (+2sd) from the nearest stem. A total of 14 sampling points were disqualified due to classification discrepancies. On average, 45% of the sample locations (min 37%, max 53%) were classified as under canopy.

#### Spatial covariates

Nine spatial covariates were computed from the DEM by considering a square kernel composed of nine grid cells with 1 m raster map resolutions. The following covariates were used in the model selection procedures at both plot and point scale: elevation, slope, aspect, northness (cosine of aspect), eastness (sin of aspect) [59], surface curvature [60], topographic position index (TPI) [61], terrain ruggedness (TRI) [62] and topographic wetness index (TWI) [63]. At a plot scale, TPI was computed relative to the surrounding 100 m. TPI is an estimate of the relative topographic position of a given point as the elevation difference between this point and the mean elevation within a set neighborhood. Positive TPI values indicates that the plot was located higher than the surrounding landscape, and negative values indicating that the plot was situated lower than the surrounding landscape. Plot values were found by averaging all cells located inside each plot, while covariates for individual sampling points were extracted from the corresponding cell.

### Modeling forest C stocks using lidar data

#### Model selection

We analyzed the use of lidar metrics against field data on several different levels: (1) Stepwise multiple regressions (Eq 2) were used to examine how the variability of C stocks in of forest compartments (mean plot values of above-ground tree, belowground tree, field layer, mosses and organic layer) at a plot level can be explained using combinations of lidar metrics as predictor variables, (2) we determined the correlation between point specific topographical values derived from lidar correlated with individual samples of field layer, mosses and organic layer C stocks for each plot and in an overall model. Assumptions of linearity, independence of errors, homoscedasticity, and normality of residuals were controlled for using standard statistical methods. Residuals deviating more than ±2 sd was assumed registration errors and excluded from further analysis. To control collinearity among the selected models, any model with variance inflation factor (VIF) larger than 10 was rejected.

$$y=b_{0}+b_{1}x_{1}+b_{2}x_{2}+\varepsilon$$ (2)

where y represents the selected forest C pool, b_0_ is the constant; b_1_, b_2_ represents the regression coefficients of the best fit model; x_1_ and x_2_ are explanatory lidar variables representing metrics of overstory, understory or local topography, and *ε* is a normally distributed error term.

As no independent data was available to assess the accuracy of the plot scale models, we used a leave-one-out cross-validation. For each step in the validation procedure, one sample plot was removed from the dataset and the selected models were fitted to the remaining plots. The goodness of fit was evaluated by root mean square error (RMSE), adjusted R^2^ and absolute bias (AB) [64].

## Results

### Carbon pools

**Fig 3:**
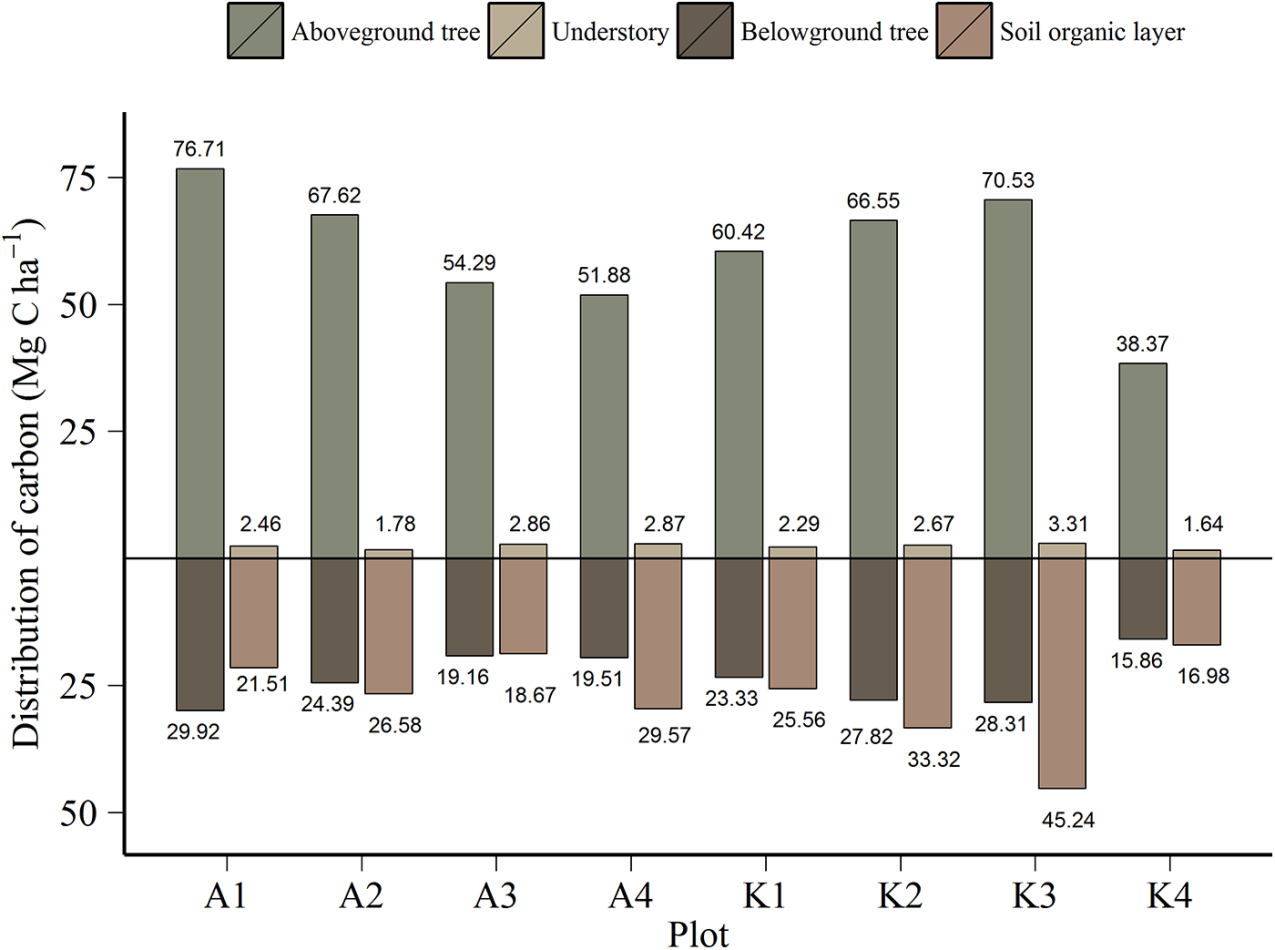
Size and distribution of C stocks by forest compartment. Distribution of C stocks (Mg C ha^-1^) by compartment at eight boreal forest plots. The understory compartment consists of field layer vegetation, mosses and saplings.

Total measured C stocks summed for all forest compartments in the eight study plots ranged from 72.85 to 147.39 Mg C ha^-1^ (Fig 3). From 48 to 59% of the C stock was found in the above-ground tree component, 19 to 23% was located in the tree roots, while 2 to 3% in the understory compartment, composed of field layer vegetation, saplings and mosses. The organic layer contained 16 to 31% of the measured C stock in these forests.

### Tree C stocks

**Fig 4:**
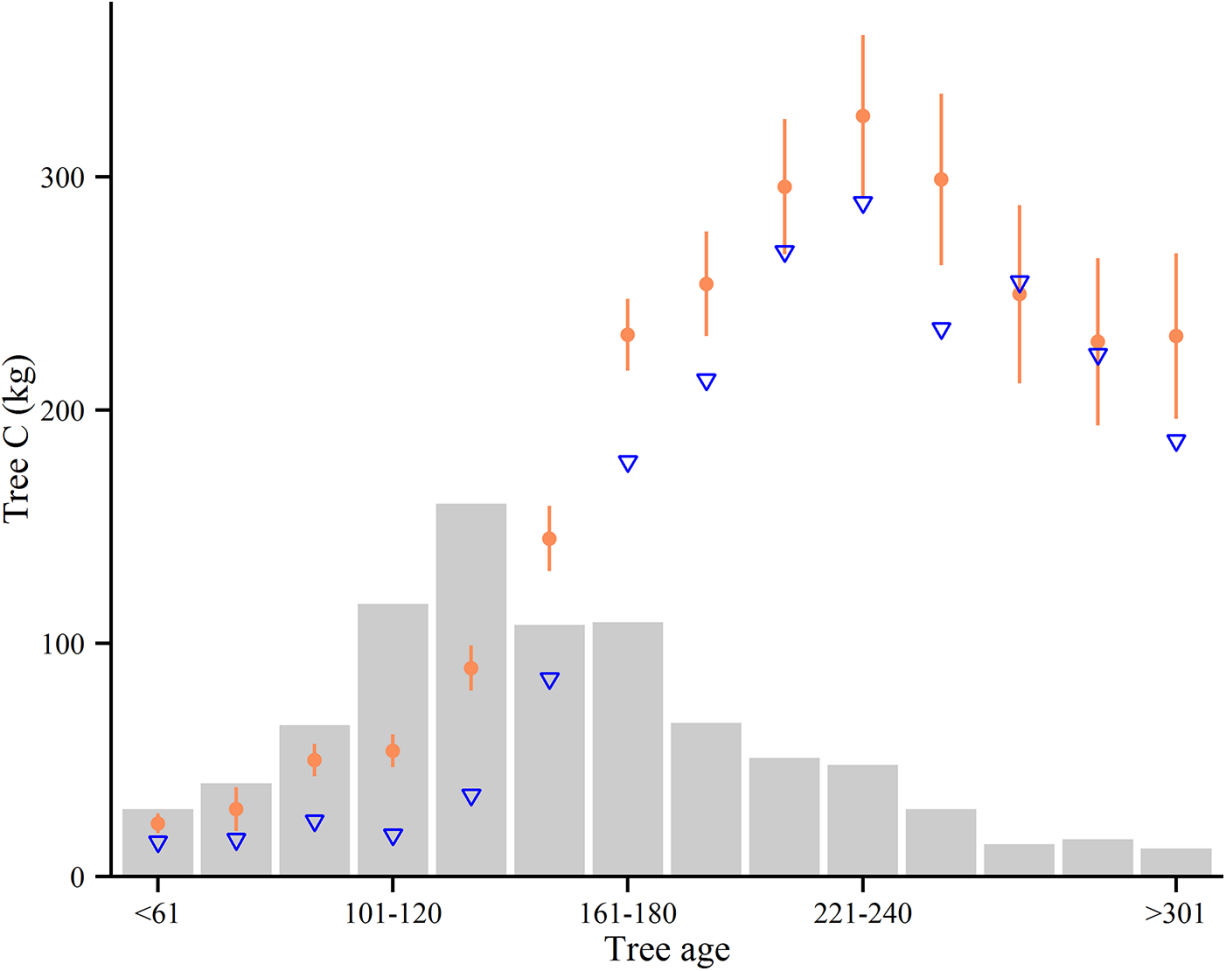
Individual tree age and C stock. The relationship between mean tree C stocks (filled circles) and median (triangles) for age groups (n = 805). Error bars indicate 95% confidence interval around mean. Bars indicate number of stems in each age bin.

The number of trees per ha^-1^ varied from 270 to 688 (Table 1), containing a total tree C stock ranging from 54.23 to 106.63 Mg C ha^-1^ (Fig 3). From 30 to 47% of the estimated tree C were found in the stems, while branches and needles accounted for 22 to 33% (data not shown). The fraction of root (> 2 mm) C in total tree C, 26 to 30%, reveals the importance of reporting the below ground tree compartment when presenting estimates of forest C. There was a negative correlation between mean plot dbh and stem density (r_(8)_ = -0.96 (95% CI: -0.79, -0.99), p < 0.001), with lower stem densities in stands with higher mean stem circumference. We were unable to associate above- and below-ground tree C stock with stem density (C_a_ r_(8)_ = -0.48 (-0.89, 0.34), p = 0.23, C_b_ r_(8)_ = -0.57 (-0.90, 0.22), p = 0.14), and mean stand age (C_a_ r_(8)_ = -0.47 (-0.89, 0.34), p = 0.24, C_b_ r_(8)_ = -0.33 (-0.84, 0.49), p = 0.43). On an individual tree basis, tree age was positively correlated with both dbh (r_(805)_ = 0.48 (0.42, 0.53), p < 0.001) and tree C (r_(805)_ = 0.38 (0.32, 0.44) p < 0.001) (Fig 4).

#### Understory C stocks

**Fig 5:**
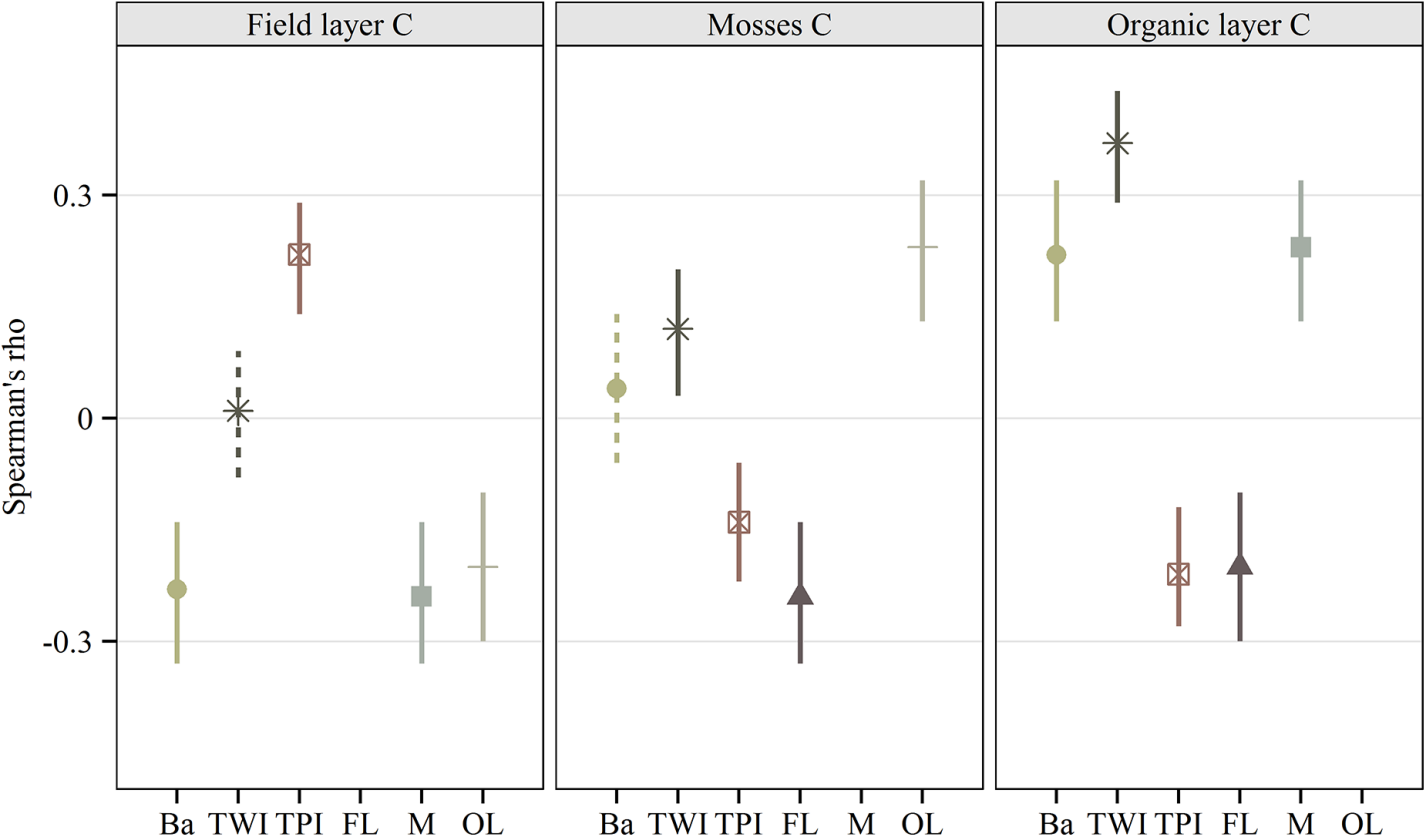
Overall correlation between individual sample characteristics. Spearman rank correlation (two-tailed) between C compartments and attributes of individual sampling point. Full lines indicate significant correlations (p < 0.01), while dotted lines show non-significant correlation (p > 0.01). Basal area (Ba) is computed in a radius of 5 m around each of the sampling points (n = 379). Topographic position index (TPI) and topographic wetness index (TWI) are derived from an interpolation of lidar ground echoes on 1 m^2^ grid cells (both n = 577). The figure also shows correlation between individual C compartments, where FL = Field layer C (n = 580), M = Mosses C (n = 583) and OL = Organic layer C (n = 556). Correlations coefficients are displayed with 95% CI determined by bootstrapping 1000 random trials for each dataset.

**Fig 6:**
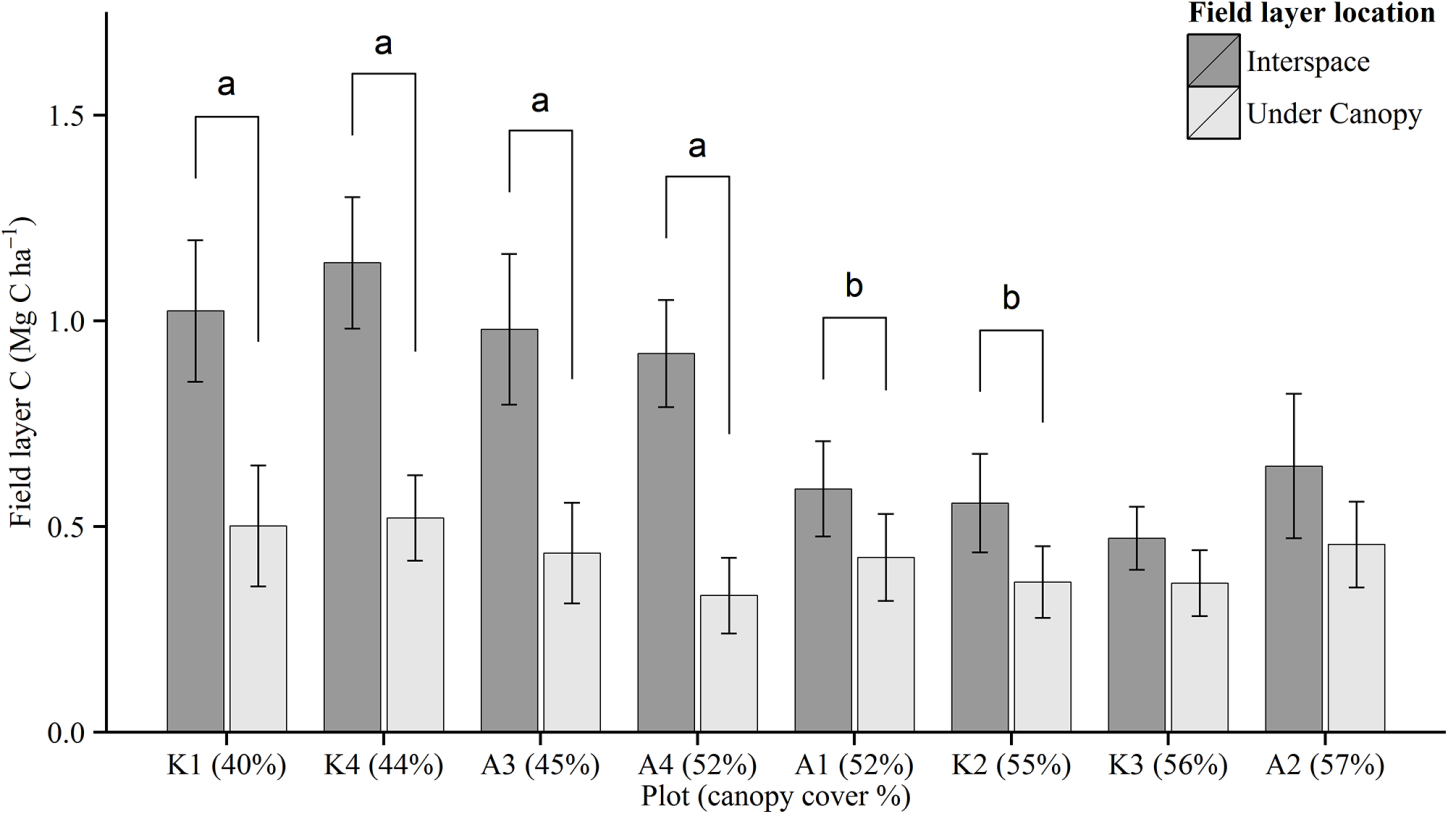
Distribution of field layer C stock by canopy cover. Bars show average plot values for the field layer C stock measurements at sampling points located under and in canopy interspace. Plots are ranked by relative canopy cover (% values) derived from lidar canopy density (cd_0_). Whiskers indicate 95% CI for the mean. Differences in mean values are indicated by significance ^a^ (p < 0.01) and ^b^ (p < 0.05).

The field layer, mosses and saplings, which comprise the understory compartment, were analyzed individually. Mean field layer C stocks varied from 0.41 to 0.88 Mg C ha^-1^ (Fig 3), with coefficients of variation (CV = sd/mean*100, %) within each plot ranging from 58 to 75%. The variances in the field layer C stocks between plots were heterogenic (Levene's test, p < 0.001), while differences were statistically significant, Welch's F (7, 243) = 13.03, p < 0.001. There was a negative correlation between plot basal area and field layer C stock (r_(8)_ = -0.82 (-0.97, -0.27), p = 0.012). The influence of trees on the field layer C stock was further investigated on a point scale using a nearest neighbor analysis and neighbor densities. We found that models of tree density performed better when including a measure of tree size, such as basal area, rather than models with only stem density. Significant trends were then observed between basal area density (in a radius of 5 m around each sampling point) and the field layer C stock in the overall model Spearman’s rho (ρ_(580)_ = -0.23 (-0.33, -0.14), p < 0.001) (Fig 5), and in six of the eight plots (results not shown). A pairwise t-test revealed significantly higher field layer C stock at sampling points situated at canopy-interspace locations than under canopy in six of the eight plots (Welch's F (1, 509) = 140.345, p < 0.001, Fig 6).

Mosses C stocks ranged from 0.76 to 2.52 Mg C ha^-1^, and were highly variable within each plot (CV 55 to 97%). The amount of mosses C was statistically different between plots, Kruskal-Wallis $\chi_{\left(7\right)}^{2}$ = 109, p < 0.001. We could not associate the mosses C stock with any of the stand attributes. The relationship between mosses and field layer C stocks at a plot level was inconclusive (r_(8)_ = -0.63 (-0.93, 0.13), p = 0.11), while the overall model indicated a significant negative correlation, Spearman’s rho (*ρ*_(570)_ = -0.17 (-0.22, -0.12), p < 0.001) (Fig 5).

Saplings numbered from 21 to 163 per plot, and never contributed more than 0.1 Mg C ha^-1^ to the understory compartment. We could not associate sapling properties with any of the stand attributes.

#### Organic layer C stocks

Mean values ranged from 16.98 to 45.24 Mg C ha^-1^ (Fig 3) and were statistically different between plots, Kruskal-Wallis $\chi_{\left(7\right)}^{2}$ = 105, p < 0.001. There were large variations in the amount of organic layer C within each plot (CV 31 to 84%), all which exceeded the intra-plot variation (CV 22%). Differences between high and low values commonly ranged from 4 to 20 times the minimum value. At one location, plot K3, the distribution was highly right-skewed, as the south-west corner of the plot was located in a transition zone between forest and peatland. Here we measured maximum values ~100 times larger than the minimum value.

The organic layer C stock was positively correlated with moss C stock at plot level (r_(8)_ = 0.74 (0.07, 0.95), p = 0.03) and in an overall model, Spearman’s rho (*ρ*_(556)_ = 0.24 (0.16, 0.32), p < 0.001) (Fig 5). When grouped by plot, the correlation was only significant in two plots (results not shown). Besides the association with mosses, we could not detect any significant relationship between the organic layer C stocks and plot attributes. However, an overall model indicated a positive relationship between individual measurements of organic layer C stock and basal area density, Spearman’s rho (*ρ*_(379)_ = 0.22 (0.12, 0.31), p < 0.001) (Fig 5). Analyzed separately, the relationship was significant at only four of the study plots (results not shown).

### Lidar data in forest C assessments

After assessing the field measured compartmental C stocks and their relationship, we then associated the data to lidar derived forest metrics. We investigated the use of lidar variables as predictors for the different forest C compartments, and analyzed the potential scale effects on the relationship of: (1) lidar metrics of above-ground stand characteristics and mean plot values of C components, (2) point specific topographical data derived from lidar and individual sampling points at individual plots and in one overall model.

#### Lidar and tree C stock

The selected models for above- (C_a_) and below-ground tree C (C_b_) stock contained a variable relating to canopy density, and h_90_, representing the 90^th^ percentiles of the canopy heights. The model for C_a_ explained 94% of the variability, whereas the model for C_b_ explained 76% of the model variability (Table 2). The selected regression model for above-ground tree C stock revealed that both maximum height h_max_ and h_90_, could be used to achieve similar explanatory power. Although the absolute difference was minimal (R^2^ and standard deviation of residuals), h_90_ had a slightly smaller standard deviation of residuals and was thus selected for the final model. Canopy density did not add any significant value to the model for below-ground C. Partial r-values for above-ground C were 0.81 (h_90_) and 0.28 (cd_0_). The variance inflation factor (VIF) was < 1.5 in both models, so multicollinearity was not considered an issue.

**Table 2:** Regression models.

	Model (Mg C ha^-1^)				
Variable	Tree C_ab_	Tree C_bel_	F-layer C	O-layer C	Plot C
Intercept	-30.68^b^	-11.48	1.97^a^	-207.99^a^	-247.37
h_90_	3.30^a^	1.12^a^			4.88^a^
cd_0_	59.93^b^	31.01	-2.66^a^		
TWI				29.00^a^	34.03
R^2^	0.95	0.82	0.83	0.75	0.79
Adj—R^2^	0.94	0.76			0.71
RMSE	3.38	2.47	0.08	4.88	12.49
BA	0.41	0.04	0.01	0.78	0.96

^a^ term is significant at the 0.01 level,

^b^ term is significant at the 0.05 level. Tree C_ab_ indicates tree C stock above ground, while Tree C_bel_ indicates tree C stock below ground.

Overall, the agreement between the models was good; above-ground tree C (r_(8)_ = 0.97 (0.84, 0.99), p < 0.001); below-ground tree C (r_(8)_ = 0.90 (0.57, 0.98), p = 0.002). The mean differences between the observed and modeled above-ground tree C ranged from -5.33 to 3.01 Mg C ha^-1^, with a corresponding SD of the differences of 2.77 Mg C ha^-1^ (4.9%) for the above-ground tree C, and -3.79 to 2.83 Mg C ha^-1^ with SD of 2.00 Mg C ha^-1^ (8.5%) for below-ground tree C.

### Lidar and understory C compartments

**Fig 7:**
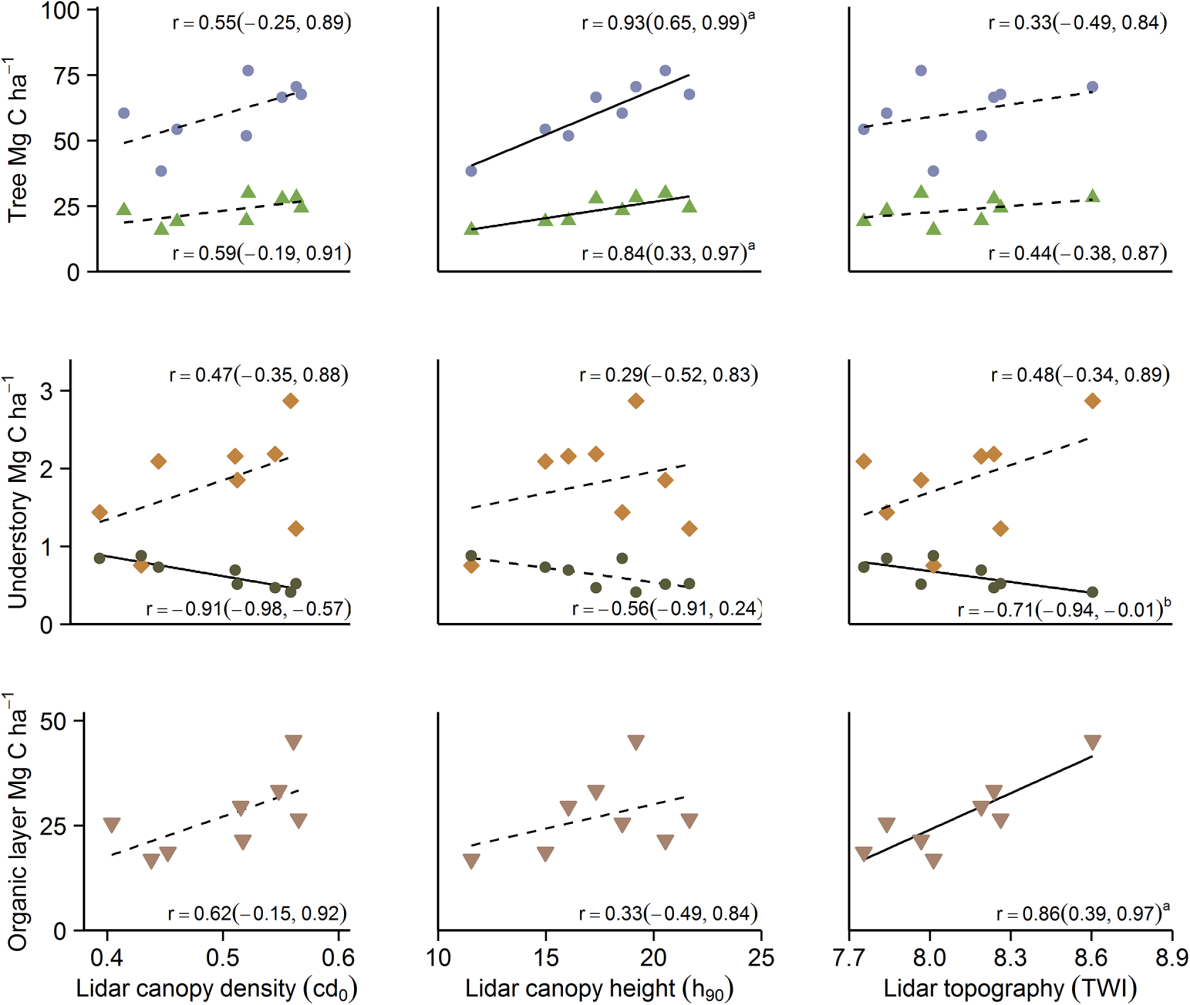
Relationship between lidar plot data and forest C compartments. Pearson’s correlation (two-tailed) of five forest C compartments and three lidar variables for the plots used in this study (n = 8). Top row show aboveground (filled circles) and belowground (filled triangles) C stocks, second row show mosses (filled squares) and field layer (filled circles) C stocks, while bottom row represent organic layer C stocks (filled triangles). Full line indicate a significant correlation (level of significance is indicated by subscript letter (*a*: p < 0.01, *b*: p < 0.05), while the dotted line show non-significant results.

The observed negative association between field layer C and basal area at a plot scale was also captured by a lidar data model using canopy density, explaining 83% of the variability (Table 2, Fig 7). Adding a buffer of 2 m for the computation window did not improve the model notably, while further expansion of the canopy computation window reduced the model fit (results not shown). Other lidar metrics such as canopy height measurements did not provide any significant correlation with the field layer C stock (Fig 7). Overall, the agreement between the model and field data were good; field layer C (r_(8)_ = 0.91 (0.61, 0.99), p = 0.001). The mean differences between the observed and modeled field layer C ranged from -0.10 to 0.08 Mg C ha^-1^, with a corresponding SD of the differences of 0.07 Mg C ha^-1^ (10.2%) for field layer C.

The proportion of understory echoes from non-canopy echoes (understory plus ground echoes) ranged from 0.19 to 0.28, with a U_i_ ranging from 0.7 to 1.7 echoes/m^2^. Mean understory heights ranged from 0.31 to 0.39 m. A total of 56 variables representing understory echoes, such as height quantiles and densities were tested against the field layer C stock situated in canopy interspace (S2 Table), but no consistent association could be determined. Similarly, the variability in sapling numbers or C stock could not be explained by any of the above-ground or topographical lidar data at a plot level.

The variability in moss C stock could not be explained by any of the above-ground or topographical lidar data at a plot level (Fig 7). When individual sampling points were used in an overall model, we detected a significant positive correlation between the moss C stock and TWI, Spearman’s rho (*ρ*_(583)_ = 0.15 (0.07, 0.23), p < 0.001) and a negative correlation between moss C stock and TPI score, Spearman’s rho (*ρ*_(583)_ = -0.13 (-0.05, -0.21), p = 0.003) (Fig 5).

### Lidar and organic layer C

At a plot level, we discovered a positive relationship between mean stand values of TWI and organic layer C, explaining 75% of the model variation (Table 2), with larger C stocks in plots with higher TWI values (Fig 5). None of the above-ground stand characteristics added any significant explanatory power to the model. Overall, the agreement between the model and field data for organic layer C was good (r_(8)_ = 0.87 (0.43, 0.98), p = 0.005). The mean differences in between the observed and modeled organic layer ranged from -6.15 to 7.49 Mg C ha^-1^, with a corresponding SD of the differences of 4.25 Mg C ha^-1^ (15.6%) for organic layer C. Similarly, there was a negative association between plot TPI and the organic layer C stock (r_(8)_ = -0.78 (-0.17, -0.96), p = 0.023), with larger organic layer C stocks in stands located in areas that are lower than the surrounding landscape. Modeling TWI and TPI with organic layer C stocks by individual points was significant in the overall models (Fig 5), but inconclusive when investigated plot by plot (results not shown). We also conducted a Mann-Whitney U test to determine if there were differences in organic layer C stocks between the two canopy classes (under–interspace). Distributions were similar, as assessed by visual inspection. Overall, the organic layer C stock was higher in sampling points under canopy (Median = 2.44, n = 253) than in canopy interspace (Median = 2.29, n = 298), U = 32767, z = -2.65, p = 0.008. However, when individual points within each plot where investigated separately, the results were inconclusive.

## Discussion

This study explored the use of lidar data beyond just quantifying tree C stocks, and found evidence that lidar canopy data can be extended to model field layer C stocks, while topographical attributes derived from lidar ground returns provides valuable indicators of the mosses and soil organic layer C stock. Attempts to increase the spatial resolution of these relationships from plot scales to 1 m^2^ cells within plots were to a large extent unsuccessful.

### Stand characteristics and C stock

#### Stand characteristics

On a local scale, the standing biomass and thus the tree C stock vary with site factors and stand properties such as tree age, stem density and species. The stands in this study have grown undisturbed from active forest management for at least 100 years [43], and the majority of trees in the canopy layer are more than 120 years old, considerably higher ages as compared to a typical managed forest stands in Norway. Mean and median dbh was negatively correlated with the number of stems, likely a result of density dependent mortality leading to self-thinning [65]. Trees accounted for ~ 97% of C in living biomass, with the majority (68 to 72%) in above-ground tree compartments, which corresponds well with previous studies of mature boreal forest stands [66, 67]. By not including the C pool in fine roots (< 2 mm), a small portion (2 to 3%) of the tree C stock [68] was left out of the assessment. However, because an estimated 10 to 40% of fine roots are located in the organic layer [68, 69], a portion of this C compartment may have been included in the soil C data.

#### Predicting tree C stocks using lidar

In this study, we were able to explain 76 to 93% of the variability in the below and above-ground tree C stock using regression models relating lidar metrics of tree height and canopy density. Our findings support previous studies showing that tree and canopy height [8, 9, 70, 71], stand basal area [6, 20] and stand volume [6, 70] and can be accurately estimated using lidar measurements, often with more accuracy than traditional assessments [71, 72].

Lidar measures of height were closely correlated to above- and below-ground tree C. Because bigger trees contribute a larger proportion of the C stock, indices reflecting height traits, should be most correlated [73]. Models containing information about large trees, such as h_90_, will weight these trees more than other measures, for example mean heights, resulting in better model fit. Lidar has long been acknowledged as a strong tool for mapping above-ground biomass [70, 73], and models with comparable explanatory power as those seen from our plots have been reporter across a broad variety of boreal forests characteristics [12, 74]. Because the aim of this study was to explore the use of lidar in mapping variations between plots with comparable ecological traits, all plots were located in the same geographic region. In large scale studies on boreal forests, the geographical region have been found to explain 32 to 38% of the variability [12], which emphasizes the importance of local sample plots for calibrating regression equations.

The estimated root compartment, which accounted for approximately a quarter of the measured tree C stock and one fifth of the measured C in these plots, highlights the importance of including roots in forest C assessments. To our knowledge, only two previous studies have reported lidar models of below ground biomass and C stock from boreal forest stands, both with comparable results [12, 75]. Assuming the general validity of the allometric equations used [52], there is no reason to believe that our predictions of root C are less valid than the above-ground estimates, showing that also the root compartment can be accurately estimated using lidar data.

Although estimation of dead woody debris was outside of the scope of this study, it may contain a substantial amount of above-ground C in mature boreal forests [76]. The C stock in dead wood have been estimated at 7.9 ±7.5 Mg C ha^-1^ across the boreal landscape, and is therefore an integral component in a full forest C inventory [77]. Several studies have reported on relationships between the variability of dead woody material and stand structural properties [76, 78], and attempts have been made to associate this compartment with structural information derived from remote sensing [79].

### Understory C and lidar

While the majority of published work on the C dynamics and stock of the boreal forest have focused on the role of trees and soils, less is known about the role and magnitude of the understory vegetation. The amount of C stored in the understory vegetation is low (2 to 5%) compared to the total above-ground C stock. These figures correspond well with previous findings from studies in boreal forest locations [32, 66]. Regardless of its relatively small contribution to forest C stock at a given time, it can be an important source of fresh biomass C (and nutrients) to the soil [31–33]. The productivity in the understory vegetation depends on a number of factors, such as e.g. light availability [80], nutrient availability [33] and the activity of the soil microbial community [18, 81]. Self-replacement through gap-fill dynamics, gives older unmanaged forests a more multi-layered characteristic than even-aged second-growth forests [80]. The heterogeneous understory growth observed in our plots is likely a result of limited and patchy light availability. As the canopy closes throughout stand development, understory vegetation shifts from early successional species like herbs and grasses towards more species adapted to low light environments, like mosses growing on the forest floor [82]. Structural changes in older stands can cause a reduction in the leaf area index, resulting in a higher relative contribution of understory vegetation to the overall net productivity [83]. Thus, stand age has been found to be a reasonable predictor of understory C storage across landscapes [31]. Although we could not reach a similar conclusion, the limited age span in our stand data (115 to 190 years) was likely inadequate for an analysis across stand development stages.

The amount of understory vegetation in boreal forests has been found to decline with increasing basal area [24, 36]. When the understory compartment is classified as one single layer, our data and results from previous studies questions the generality of those conclusions [31]. As the C stocks of field layer and mosses were negatively correlated, models improved by treating the two compartments separately instead of aggregated in a single (understory) compartment. While no conclusions could be drawn from the relationship between above-ground structures and the mosses C stock, we observed a negative association in both absolute and relative amounts of field layer C (as a proportion to the understory C stock) under stands with higher basal area. Lower light conditions in denser canopies will likely limit growth of shade intolerant species such as *V*. *myrtillus* [84], which were commonly observed on all plots in this study. A negative correlation between stand basal area and field layer biomass in spruce, pine and broad-leaved forest stands have also been reported from Finland [31]. When the resolution of basal area was increased from a single value representing plots to the basal area density in a 5 m radius of individual sampling points, the relationship to field layer C stock became more diffuse. However, these models were slightly improved by fitting the density of above-ground biomass instead of basal area. While basal area is a function of dbh squared, biomass is a product of wood density and stem volume (basal area and height), and will therefore increase as a function of dbh to a power greater than 2 [73]. The improved model fit when shifting from basal area to above-ground biomass might therefore be explained by the effects of larger trees within each stand [85].

The literature associating lidar data directly with understory characteristics are rather scarce. Structural parameters from lidar have successfully been used to identify site class [86], map distribution of mosses [40] and lichen in boreal conifers [87], understory species composition in temperate deciduous woodlands [39, 88] and subalpine conifer dominated forests [41]. Other studies have found lidar to provide accurate estimations of leaf area index [37, 89] and canopy gaps [90], both which affects light transmittance to the ground. Because light availability is considered the main limiting growth factor for field layer vegetation [35, 91], canopy density measures from lidar have shown to be good proxies for understory light availability [38]. When the understory was investigated separately, our results indicate that on a plot scale, canopy density measures from lidar were better predictors of field layer C stock than any of the stand measurements collected in the field. Similarly, the effect of the canopy on field layer C stock was also observed at a point scale where samples were classified by their location in canopy gaps or under canopy. Studies of shrub presence in both young and mature mixed conifer forests in Northern Idaho, USA have reported similar findings [42]. However, it is worth noticing that our field layer C stock is 2.5 to 5.6 times smaller than the standard deviation of predicted above-ground tree C. Although the limited number of observations in this study prevents development of regression models with a large generality, the possibility that a significant correlation could be detected among plots of comparable structural characteristics is encouraging, and suggests that further investigation across a wider range of ecological variables could yield valuable insight.

Even though the field layer C stock in canopy gaps was dissimilar between plots, we could not find evidence associating any lidar understory metrics to this variability. Computation of understory lidar metrics were done by applying different filters and classifications, but only marginal differences in height indices were found. The poor fit between understory returns and the field layer might be a result of a low pulse density and the ability to differentiate among small height classes [92]. Thus, errors may occur where the ground vegetation is dense, making it difficult to separate ground hits from low vegetation [27, 92]. It may also be a result of unsuitable plot area (~2000 m^2^), as neighboring trees will have a greater influence on light conditions than more distant ones. Relationships between canopy structure derived from lidar and understory growth have shown to be scale dependent, and more precise models have been found for 225 m^2^ plots, than 25 m^2^ and 100 m^2^ plots [93].

### Organic layer C and lidar

The organic layer in boreal forest commonly displays great spatial variability [15, 16, 94], with differences between minimum and maximum values up to 100 times over relatively short distances, making precise estimates of the organic layer C stock inherently challenging. The complex configuration of organic layer C is not a result of linear and additive set of causes, but is instead affected by a range of interrelated environmental variables, each with a number of potential effects, making precise mapping and monitoring soil C a formidable task.

Tree species have long been recognized as an important factor for organic layer dynamics [29], affecting the properties by root activity, microclimate and chemical constituents in the litter [19, 95]. Although the relative contribution of different tree compartments (roots, leaves) to the soil organic C pool varies between species and local conditions [34], the link between above and below-ground processes are strong [23, 81]. Stand basal area, which can be derived from lidar data [6], have also been associated with soil C storage [24]. We found comparable results between basal area densities and the organic layer C stock in only three out of the eight plots used in this study. However, when scaled from single sampling points up to mean plot densities, this association was no longer detectable. This might be a result of the relatively low number of plots in this study, or that such patterns might only be observable when compared across a wider range of age classes.

Due to quality differences in plant residue and mineralization rates among deciduous and conifer stands [96], and even between spruce and pine [24, 25], observed differences in organic layer C stock have been linked to the local dominating tree species [97]. In particular, studies have found the organic soil layer under spruce stands to be both thicker and have a higher C stock than those of pine and birch [24, 25]. Whether these observed differences are due to tree species effects is questionable, since trees themselves are not randomly distributed in natural forests, but follow gradients in climate, successional stage, soil type and other abiotic factors [97]. Because the forest stands used in this study were heavily dominated by Norway spruce, we were unable to test this hypothesis. Nevertheless, in boreal forests, which consist of relatively few tree species, lidar has successfully been used to predict species of individual trees. The overall classification accuracy is high, ranging from 88 to 96% [21, 22].

Because soil attributes have been linked to the local topography [26, 30], another promising aspect of lidar for assisting in mapping soil organic C is the capacity to provide fine scale topographical information from interpolating ground echoes. The absolute accuracy of the DEM is a function of sampling density, scanning angle, canopy density and closure [27, 98]. In the boreal zone, under the right conditions, terrain models with random errors less than 20 cm can be obtained [27]. Due to the internal consistency of lidar elevation data, topographical variables derived from DEM products, such as slope and aspect, usually have higher accuracies than the lidar elevation data [99]. For the purpose of mapping soils, accuracy can be further enhanced by combining DEM and local soil data using soil mapping methods [26]. Depending on the existing soil information, terrain attributes from lidar can be used as a soil covariate in an interpolation approach filling in gaps in the soil map [100]. For predictions of soil or mosses C stocks, topographical features which are particularly interesting are the local elevation, slope, and concavity, as these influences the local hydrological conditions and thus the C accumulation rate [101]. Secondary indexes computed from elevation and slope, TWI and TPI, which were associated to the organic layer C in our plots, have been used in earlier studies to estimate soil attributes and C stocks [28, 102].

In this study all survey plots were purposively located under forest cover, and our estimates may therefore not be a good indication of the mean C stock across the landscape as a whole, but should instead be considered a mean estimate of a stratum within a landscape. As the primary motivation for investigating the use of lidar in soil C mapping is to enable assessments over across larger landscapes and regions, we would recommend that future studies include a broader range of ecological variables and soil types into their models. Linking lidar data with ground plot data can improve the feasibility of mapping and monitoring soil C stocks across larger regions, by aiding the surveyor in the stratification and layout of sampling plots.

Despite the ability to delineate high resolution data on topographical variables, there have been very limited attempts to directly associate boreal soil C stock with lidar data. At a plot scale, our results indicate that the variability in both organic layer and mosses C stocks can be partly explained by topographical variables, such as TWI and TPI. However, when the resolution is enhanced from single plot averages to 1 m^2^ grid cells within the plots, the correlation becomes increasingly diffuse. This could be a consequence of spatial inaccuracy in field measurements or a lacking ability to differentiate between ground returns and biomass in areas with dense understory vegetation, masking some of the fine-scale topographical features. Technological improvements, such as full waveform systems, increased laser density, and inclusion of other ecological variables into the model may enhance the predictive power.

## Conclusion

The high resolution sampling design employed in this study, combined with lidar data acquisition, provide a unique opportunity to examine the association between stand structure characteristics and C pools in different forest compartments across different scales. Overall, compartmental C models at plot scale (~2000 m^2^) performed better than models with higher resolution (1 m^2^). We find that canopy density and height metrics derived from lidar can be used to accurately estimate both above and belowground tree C, while canopy density measures alone are strong predictors of the field layer C stock. We also demonstrate that the organic layer C stock can be modeled with good accuracy using topographical characteristics derived from lidar ground echoes. Depicting whole plot C storage from a universal equation did not yield significant results, due to the dominance of tree C storage and the lack of association between drivers of C stocks in soils and trees.

Attempts to associate echoes directly from the field layer with the C stock in this compartment were unsuccessful. Similarly, efforts to associate individual soil C sampling points to local terrain characteristics by increasing the resolution from a plot level to 1 m^2^ did not yield consistent results, possibly due to the low echo density in areas under canopy. Increasing the laser density, using full waveform systems, or incorporating other remote sensing techniques, such as hyper-spectral imagery may improve explanatory power for predicting understory conditions and fine details in topography. In particular, recent advances in the use of full waveform lidar, which digitize and record the entire backscattered signal of each emitted pulse, can enhance the ability to characterize overstory structures, and thus improve predictions of compartmental C storage.

In the search for an effective tool to measure and monitor forest C pools, we find the capabilities of lidar encouraging. The methods employed in this study warrants further investigation across a wider range of ecological variables and scales.

## Supporting Information

S1 Table Biomass functions. (PDF)

S2 Table Lidar variables. (PDF)
