# Supplementary material for: Mapping Above- and Below-Ground Carbon Pools in Boreal Forests: The Case for Airborne Lidar
Source: PLoS One. 2015 Oct 1;10(10):e0138450. doi: 10.1371/journal.pone.0138450 (PMC4591287; doi:10.1371/journal.pone.0138450)
Supplement: S1 Table — (PDF) [file pone.0138450.s001.pdf]

S1 Table. Biomass equations

Derived from Marklund (1988) and Peterson and Ståhl (2006).

**Norway Spruce (*Picea abies*)**	
Stem + Bark	Ln (S+B) = -2.1702+7.4690*d/(d+14)+0.0289*h+0.6828*ln(h)
Branches and needles	Ln (B+N)= -1.2063+10.9708*d/(d+13) -0.0124*h-0.4923*ln(h)
Stumps	Ln (S) = -3.3645+10.6686*d/(d+17)
Roots (< 5mm)	Ln (R) = 4.52965+10.57571*d/(d+142)
**Scots Pine (*Pinus sylvestris*)**	
Stem + Bark	Ln (S+B) = 2.6768 + 7.5939*d(d+13)+0.0151*h+0.8799*ln(h)
Branches and needles	Ln (B+N) = 2.8604 + 9.1015*d/(d+10)
Stumps	Ln (S) = 3.3913+11.1106*d/(d+12)
Roots (< 5mm)	Ln (R) = 3.390+11.068*d/(d+113)
**Birch (*Betula pendula, Betula pubescence*)**	
Stem	Ln (S) = -3.5686 + 8.2827*d(d+7)+0.00393*h+0.5772*ln(h)
Branches	Ln (B) = -3.3633+10.2806*d/(d+10)
Leaves	Ln (L) = -3.9823+8.058*d/(d+8)
Roots (< 5mm)	Ln (R) = 4.909+9.912*d/(d+138)

d = diameter at breast height (cm), h = height (m). The results are returned in kilograms of dry weight. Roots are returned in grams.
