# Supplementary material for: Mapping Above- and Below-Ground Carbon Pools in Boreal Forests: The Case for Airborne Lidar
Source: PLoS One. 2015 Oct 1;10(10):e0138450. doi: 10.1371/journal.pone.0138450 (PMC4591287; doi:10.1371/journal.pone.0138450)
Supplement: S2 Table — (PDF) [file pone.0138450.s002.pdf]

S2 Table. Lidar variables

Note: To explore the use of lidar data in mapping and quantifying forest C stocks we used echoes from the *first* returns only and a combination of *both* first and last returns. *From* represent the cutoff height or bin range (in m) of lidar echo.

Id	From	Name	Short	First/combined	Reference/Note
1	Field	Basal area	Ba	Both	
2	Lidar ground	Elevation	Elev	Both	
3	Lidar ground	Slope	Slope	Both	
4	Lidar ground	Aspect	Aspect	Both	
5	Lidar ground	Slope Degrees	SlopeDeg	Both	Stage (1976)
6	Lidar ground	Aspect Degrees	AspectDeg	Both	Stage (1976)
7	Lidar ground	Topographic Ruggedness Index	TRI	Both	Riley et al. (1999)
8	Lidar ground	Topographic Position Index	TPI	Both	Guisan et al. (1999)
9	Lidar ground	Curvature	Curvature	Both	Zevenbergen and Thorne (1987)
10	Lidar ground	Topographic Wetness Index	TWI	Both	Gessler et al. (1995)
11	Lidar Overstory	Height Percentile_{10}	h_{10}	First	
12	Lidar Overstory	Height Percentile_{20}	h_{20}	First	
13	Lidar Overstory	Height Percentile_{30}	h_{30}	First	
14	Lidar Overstory	Height Percentile_{40}	h_{40}	First	
15	Lidar Overstory	Height Percentile_{50}	h_{50}	First	
16	Lidar Overstory	Height Percentile_{60}	h_{60}	First	
17	Lidar Overstory	Height Percentile_{70}	h_{70}	First	
18	Lidar Overstory	Height Percentile_{80}	h_{80}	First	
19	Lidar Overstory	Height Percentile_{90}	h_{90}	First	
20	Lidar Overstory	Height Percentile_{95}	h_{95}	First	
21	Lidar Overstory	Height Percentile_{mean}	h_{mean}	First	
22	Lidar Overstory	Height_{CV}	h_{CV}	First	
23	Lidar Overstory	Canopy Density_{0}	Cd_{0}	First	
24	Lidar Overstory	Canopy Density_{10}	Cd_{10}	First	
25	Lidar Overstory	Canopy Density_{20}	Cd_{20}	First	
26	Lidar Overstory	Canopy Density_{30}	Cd_{30}	First	
27	Lidar Overstory	Canopy Density_{40}	Cd_{40}	First	
28	Lidar Overstory	Canopy Density_{50}	Cd_{50}	First	
29	Lidar Overstory	Canopy Density_{60}	Cd_{60}	First	
30	Lidar Overstory	Canopy Density_{70}	Cd_{70}	First	
31	Lidar Overstory	Canopy Density_{80}	Cd_{80}	First	
32	Lidar Overstory	Canopy Density_{90}	Cd_{90}	First	
33	Lidar Overstory	Stratum 1	$Strat_{1}$	First	From 1.5 - 2.5
34	Lidar Overstory	Stratum 2	$Strat_{2}$	First	From 2.5 - 5
35	Lidar Overstory	Stratum 3	$Strat_{3}$	First	From 5 - 10
36	Lidar Overstory	Stratum 4	$Strat_{4}$	First	From 10 - 20
37	Lidar Overstory	Stratum 5	$Strat_{5}$	First	From >20

38	Lidar Understory	Height Percentile$_{10}$ - Filter 1	UhF1B$_{10}$	Both	From 0.01
39	Lidar Understory	Height Percentile$_{20}$ - Filter 1	UhF1B$_{20}$	Both	From 0.01
40	Lidar Understory	Height Percentile$_{30}$ - Filter 1	UhF1B$_{30}$	Both	From 0.01
41	Lidar Understory	Height Percentile$_{40}$ - Filter 1	UhF1B$_{40}$	Both	From 0.01
42	Lidar Understory	Height Percentile$_{50}$ - Filter 1	UhF1B$_{50}$	Both	From 0.01
43	Lidar Understory	Height Percentile$_{60}$ - Filter 1	UhF1B$_{60}$	Both	From 0.01
44	Lidar Understory	Height Percentile$_{70}$ - Filter 1	UhF1B$_{70}$	Both	From 0.01
45	Lidar Understory	Height Percentile$_{80}$ - Filter 1	UhF1B$_{80}$	Both	From 0.01
46	Lidar Understory	Height Percentile$_{90}$ - Filter 1	UhF1B$_{90}$	Both	From 0.01
47	Lidar Understory	Height Percentile$_{mean}$	UhB$_{mean}$	Both	From 0.01
48	Lidar Understory	Height Percentile$_{10}$ - Filter 2	UhF2B$_{10}$	Both	From 0.2
49	Lidar Understory	Height Percentile$_{20}$ - Filter 2	UhF2B$_{20}$	Both	From 0.2
50	Lidar Understory	Height Percentile$_{30}$ - Filter 2	UhF2B$_{30}$	Both	From 0.2
51	Lidar Understory	Height Percentile$_{40}$ - Filter 2	UhF2B$_{40}$	Both	From 0.2
52	Lidar Understory	Height Percentile$_{50}$ - Filter 2	UhF1B$_{50}$	Both	From 0.2
53	Lidar Understory	Height Percentile$_{60}$ - Filter 2	UhF2B$_{60}$	Both	From 0.2
54	Lidar Understory	Height Percentile$_{70}$ - Filter 2	UhF2B$_{70}$	Both	From 0.2
55	Lidar Understory	Height Percentile$_{80}$ - Filter 2	UhF$_{2}$B$_{80}$	Both	From 0.2
56	Lidar Understory	Height Percentile$_{90}$ - Filter 2	UhF$_{2}$B$_{90}$	Both	From 0.2
57	Lidar Understory	Height Percentile$_{mean}$ - Filter 2	UhF$_{2}$B$_{mean}$	Both	From 0.2
58	Lidar Understory	Height Percentile$_{10}$ - Filter 1	UhF1F$_{10}$	First	From 0.01
59	Lidar Understory	Height Percentile$_{20}$ - Filter 1	UhF1F$_{20}$	First	From 0.01
60	Lidar Understory	Height Percentile$_{30}$ - Filter 1	UhF1F$_{30}$	First	From 0.01
61	Lidar Understory	Height Percentile$_{40}$ - Filter 1	UhF1F$_{40}$	First	From 0.01
62	Lidar Understory	Height Percentile$_{50}$ - Filter 1	UhF1F$_{50}$	First	From 0.01
63	Lidar Understory	Height Percentile$_{60}$ - Filter 1	UhF1F$_{60}$	First	From 0.01
64	Lidar Understory	Height Percentile$_{70}$ - Filter 1	UhF1F$_{70}$	First	From 0.01
65	Lidar Understory	Height Percentile$_{80}$ - Filter 1	UhF1F$_{80}$	First	From 0.01
66	Lidar Understory	Height Percentile$_{90}$ - Filter 1	UhF1F$_{90}$	First	From 0.01
67	Lidar Understory	Height Percentile$_{mean}$	UhF1F$_{mean}$	First	From 0.01
68	Lidar Understory	Height Percentile$_{10}$ - Filter 2	UhF2F$_{10}$	First	From 0.2
69	Lidar Understory	Height Percentile$_{20}$ - Filter 2	UhF2F$_{20}$	First	From 0.2
70	Lidar Understory	Height Percentile$_{30}$ - Filter 2	UhF2F$_{30}$	First	From 0.2
71	Lidar Understory	Height Percentile$_{40}$ - Filter 2	UhF2F$_{40}$	First	From 0.2
72	Lidar Understory	Height Percentile$_{50}$ - Filter 2	UhF2F$_{50}$	First	From 0.2
73	Lidar Understory	Height Percentile$_{60}$ - Filter 2	UhF2F$_{60}$	First	From 0.2
74	Lidar Understory	Height Percentile$_{70}$ - Filter 2	UhF2F$_{70}$	First	From 0.2
75	Lidar Understory	Height Percentile$_{80}$ - Filter 2	UhF2F$_{80}$	First	From 0.2
76	Lidar Understory	Height Percentile$_{90}$ - Filter 2	Uh$_{F2}$F$_{90}$	First	From 0.2
77	Lidar Understory	Height Percentile$_{mean}$ - Filter 2	UhF2F$_{mean}$	First	From 0.2
78	Lidar Understory	Density Stratum 1 - Filter 1	UdF1B$_{1}$	Both	From 0.01 - 0.5
79	Lidar Understory	Density Stratum 2 - Filter 1	UdF1B$_{2}$	Both	From 0.5 to 1
80	Lidar Understory	Density Stratum 3 - Filter 1	UdF1B$_{3}$	Both	From 1 to 1.5
81	Lidar Understory	Density Stratum 1 - Filter 2	UdF2B$_{1}$	Both	From 0.2 - 0.5
82	Lidar Understory	Density Stratum 2 - Filter 2	UdF2B$_{2}$	Both	From 0.5 to 1

83	Lidar Understory	Density Stratum 3 - Filter 2	$UdF2B_3$	Both	From 1 to 1.5
84	Lidar Understory	Density Stratum 1 - Filter 1	$UdF1F_1$	First	From 0.01 - 0.2
85	Lidar Understory	Density Stratum 2 - Filter 1	$UdF1F_2$	First	From 0.2 to 1
86	Lidar Understory	Density Stratum 3 - Filter 1	$UdF1F_3$	First	From 1 to 1.5
87	Lidar Understory	Density Stratum 1 - Filter 2	$UdF2F_1$	First	From 0.01 - 0.2
88	Lidar Understory	Density Stratum 2 - Filter 2	$UdF2F_2$	First	From 0.2 to 1
89	Lidar Understory	Density Stratum 3 - Filter 2	$UdF2F_3$	First	From 1 to 1.5
90	Lidar Understory	Proportion Non Ground - Filter 1	$UF1B_i$	Both	From 0.01 to 1.5
91	Lidar Understory	Proportion Non Ground - Filter 2	$UF2B_i$	Both	From 0.2 to 1.5
92	Lidar Understory	Proportion Non Ground - Filter 1	$UF1F_i$	First	From 0.01 to 1.5
93	Lidar Understory	Proportion Non Ground - Filter 2	$UF2F_i$	First	From 0.2 to 1.5
